# Long term outcome after 48 Gy stereotactic ablative body radiotherapy for peripheral stage I non-small cell lung cancer

**DOI:** 10.1186/s12885-019-5863-2

**Published:** 2019-06-28

**Authors:** Emilie Dubaere, Mathilde Goffaux, Marie Wanet, Benoit Bihin, Céline Gheldof, Anne-Sophie Demoulin, Antoine Bolly, Frederique Bustin, Fabrice Duplaquet, Paul-Emile Baugnee, Michel Gustin, Vincent Hers, Fabienne Maisin, Eric Marchand, Sebahat Ocak, Lionel Pirard, Oswald Vancutsem, Evelyne Van Neck, Guy Vandermoten, Luminata Zaharia, Vincent Remouchamps

**Affiliations:** 1 Department of Radiotherapy, CHU UCL Namur, Site Ste Elisabeth, Place Louise Godin 15, 5000 Namur, Belgium; 2 NARILIS, Namur, Belgium; 3 CHU UCL Namur, Site Godinne, Yvoir, Belgium; 4 CHR Citadelle Liege, Liege, Belgium; 5 CHR Sambre et Meuse, Namur, Belgium; 6 Clinique St Luc Bouge, Namur, Belgium; 7 CHU UCL Namur, Site Dinant, Dinant, Belgium; 8 Institut de Recherche Expérimentale et Clinique (IREC), Pôle de Pneumologie, ORL et Dermatologie (PNEU), Université Catholique de Louvain (UCL), Brussels, Belgium

**Keywords:** Stereotactic ablative radiotherapy, Lung cancer, Non-small cell lung cancer, Survival

## Abstract

**Background:**

To evaluate the outcome of patients treated with stereotactic ablative body radiotherapy (SABR) with curative intent for stage I non-small cell lung cancer (NSCLC) with regard to local, regional and distant tumor control, disease-free survival (DFS), overall survival (OS) and toxicity.

**Methods:**

Data of 300 patients treated with SABR for NSCLC cancer for the period of November 2007 to June 2016 were retrospectively analyzed. Of which, 189 patients had single primary lung lesion and were included in the study. The prescribed dose for the tumor was 48 Gy, given in 12 Gy × 4 fractions for all patients. In 2010, an improved protocol was established in advanced technology for the planning CT, dose calculation and imaging. Cumulative incidence function (CIF) of local, regional, distant or any recurrences were computed using competing risk analysis with death as a competing event. Survivals (DFS and OS) were estimated using the Kaplan-Meier method and Cox proportional regression was used for comparisons. Toxicities were graded according to the common terminology criteria for adverse events version 4.0 (CTCAE v.4).

**Results:**

Diagnosis was histologically confirmed in 42% of the patients (*N* = 80). At 1, 2 and 4 years, the cumulative incidence function (CIF) of local relapses were 8% [4–13%], 15% [10–21%] and 18% [12–25%], the CIF of regional relapses were 4% [2–8%], 10% [6–16%] and 12% [8–19%], the CIF of distant relapses were 9% [5–14%], 15% [11–22%] and 20% [15–28%] and the CIF of any relapses were 14% [10–20%], 28% [22–36%], 34% [27–43%], respectively. After 1, 2 and 4 years, the OS rates were 83% [95% CI: 78–89%] (*N* = 128), 65% [95% CI: 57–73%] (*N* = 78) and 37% [95% CI: 29–47%] (*N* = 53), respectively. The median survival time was 37 months. The DFS after 1, 2 and 4 years reached 75% [95% CI: 68–81%] (*N* = 114), 49% [95% CI: 42–58%] (*N* = 60) and 31% [95% CI: 24–41%] (*N* = 41), respectively. No grade 4 or 5 toxicity was observed.

**Conclusions:**

We observed a long-term local control and survival after SABR for peripheral stage I NSCLC in this large series of patients with the expected low toxicity.

## Background

The standard treatment for stage I non-small cell lung cancer (NSCLC) is surgery, with excellent local control and survival outcome [1, 2]. However, a substantial proportion of patients are unfit to tolerate any type of surgical resection due to their comorbidities. Furthermore, a small proportion of patients rejected the option of surgery based on personal reasons. The alternative treatment for these patients was no treatment at all or primary conventional radiotherapy with a total dose of 60 to 70 Gy in fractions of 2 Gy a day [3]. The outcome after conventional radiotherapy has been relatively poor, with a long term survival of 15–30% and local control only being 40–50% [4, 5]. Due to advances in radiation modalities, the management upgraded to stereotactic ablative body radiotherapy (SABR). Better local control may be reached by increasing radiation therapy dose, resulting in better overall survival (OS) [6]. SABR is a radiation technique which assigns a high radiation dose with precise delivery and high conformity while avoiding radiosensitive organs surrounding the tumor. In comparison to conventional radiotherapy, which is delivered over several weeks, SABR is typically administered in few fractions, typically 3 to 10 fractions, reducing the overall treatment time. In addition, the toxicity profile of this non-invasive treatment is quite favorable [7, 8]. An excellent clinical outcome with SABR has been observed with several studies showing comparable results to those obtained through surgery. Local control rates are approximately 80 to 90% when schedules with biologically effective dose (BED) larger than 100 Gy are used [9–12].

We retrospectively evaluated the local, regional and distant tumor control, as well as the disease-free survival (DFS), OS and toxicity in our unselected patient population that was treated over the last 10 years. In recent years, there have been advances in the radiotherapy techniques, delivery for SABR and treatment methods in our department, however, the 12 Gy × 4 fractions prescription has remained unaltered.

## Methods

### Patient population

Data of 300 patients with stage I NSCLC (American Joint Committee on Cancer, seventh edition T1a T1b-T2 N0 M0) [13] treated for peripheral lung lesions with a standard SABR protocol between November 2007 and June 2016 were retrospectively reviewed. The review was approved by the Institutional Review Board under the current legislation (Ethics Committee from CHU UCL Namur, site Ste. Elisabeth and its President, Dr. Isabelle Mathieu). The acceptance date of the ethics committee was 21 October 2016. Patients with central lesions, multiple nodules, metastatic lung lesions or synchronous cancers were excluded. Also, patients were recruited from several institutions but treated in a single radiation therapy department. In-hospital medical files and letters from the referring pneumologist or oncologist for patients followed-up on in collaborating institutions were reviewed.

An experienced multidisciplinary team carefully studied patient’s characteristics (i.e. smoking history and age) and their images before suggesting treatment without histological confirmation. There was a need for an adequate record of the growth of the tumor size over time, a high ^18^F-FDG-PET uptake and morphological abnormalities suggestive of malignancy [14, 15].

When follow-up is lost, which usually happens after supportive care, the vital status was obtained after phone calls to the family or general practitioners.

### Procedures

#### Image acquisition

All patients underwent fluoro-deoxy-glucose positron emission tomography and computed tomography (FDG-PET-CT) for staging, usually contrast-enhanced. All patients underwent planning CT with a slice thickness of 2.5 mm and a four-dimensional (4D)-CT on a dedicated CT simulator (General Electric RT16, General Electric Company, Boston). Two different 4D image acquisition procedures were used according to the date of patient inclusion in the study. Initially, a 3 phase CT including a physiological inspiration, expiration and free breathing were performed. From the end of 2009, a 10 phase 4D-CT using the Real-time Position Management (RPM®, Varian Medical Systems, Palo Alto, CA, USA) device was used. Patients were immobilized with a thermoplastic mask. In 2010, the diaphragmatic compression was added. Images were transferred to the Eclipse planning system (TPS; Eclipse version 8 to 11 during this period, Varian Medical Systems, Palo Alto, CA, USA).

#### Organs at risk and volumes delineation

The gross tumor volume (GTV) included the visible tumor, delineated with a lung windowing setting (level − 600 Hounsfield Unit (HU); width 1600 HU). An internal target volume (ITV) encompassing all tumor position in the breathing cycle was delineated using all phases of the 4D-CT. No clinical target volume (CTV) margins were used. An isotropic margin of 5 mm around the ITV was used to create the setup margin component of planning target volume (PTV).

The organs at risk (OARs), that is, the spinal cord, lungs, heart, great vessels, proximal bronchi, brachial plexus, chest wall, trachea, esophagus and skin, were delineated depending on the location of the tumor. According to reports 62 and 83 of the International Commission on Radiation Units and measurements, a safe 5 mm margin was added to the spinal cord as planning at risk volume (PRV). Adding a PRV for the esophagus or 4D delineation according to the location of the target was hardly necessary as they were all peripheral lesions.

#### Treatment planning

For all patients, the total prescribed dose to the PTV was 48 Gy in 12 Gy × 4 fractions. Treatments were administered on a linear accelerator (Clinac® 21EX; Varian Medical Systems, Palo Alto, CA, USA and Novalis TX; Varian Medical Systems, Palo Alto, CA, USA) delivering 6 MV photons. Dose constraints for OARs and target volumes (TVs) were compliant with the national protocol derived from RTOG trial 0915. From 2007 to 2010, most patients were treated with multiple (minimum of seven) coplanar and/or non-coplanar beams with three-dimensional conventional radiotherapy (3D-CRT) using pencil beam dose calculation algorithm (Type I dose computation algorithm). Twenty five percent of patients from the period before 2010 with limited breathing motion (less than 5 mm) on inspiration and expiration CT were treated on the Tomotherapy unit, while the others were treated on a 5 mm leaves Millenium multi-leaf collimator (MLC) linear accelerator. The total dose was prescribed at the isocenter, accepting 80–95% around the PTV [16]. Fractions were delivered every other day. On the Tomotherapy system, a type II calculation algorithm was used. From 2010, all patients were treated with volumetric modulated arc therapy (VMAT) with the Varian method named Rapidarc on a Novalis Tx linear accelerator using an analytic anisotropic algorithm (AAA) calculation for treatment planning (Type II dose computation algorithm) and a 2.5 mm leaves MLC. Two half arcs with different collimation angles were used to avoid interplay effect. The dose was prescribed to 80% isodose encompassing the PTV (48 Gy isodose around the PTV), with an aimed dose inhomogeneity allowing a 100% maximum dose in the GTV, corresponding to a 25% over dosage located in the GTV. This method was copied from the RTOG protocols. The later release of the ICRU report 91 confirmed these evolutions [17]. Beginning from 2010, fractions were allowed once a day with a 48 h break in the middle of the week, but most patients were treated with two to three fractions a week for logistic reasons.

#### Setup and delivery procedures

Initially, verification of patient setup and tumor position was performed daily using orthogonal Mega Voltage (MV) portal images with online correction and additional 2D-real time cine-acquisition images. On these images, the tumor and its motion could be seen in the Beam Eye View of some beam angles for most patients. Patients in this period treated on the Tomotherapy machine were positioned with on-line volumetric MV CT imaging before and during the fraction. Systematic volumetric Cone-Beam Computed Tomography (CBCT) came into the scene in 2010. It was performed before the start of the session and with a second CBCT in between two arcs in order to verify the intra-fraction stability.

#### Toxicity

Radiation-induced toxicities were evaluated according to the Common Terminology Criteria for Adverse Events (CTCAE) version 4.0.

#### Follow-up

First clinical and radiological follow-up is usually done 3 months after the completion of radiotherapy and subsequently, every 6 months. Clinical examination and chest CT with complementary FDG-PET-CT in case of uncertain recurrence were performed.

Local, regional and distant recurrences were censored. Local recurrence includes a failure within or adjacent to the PTV. Local control was defined as the absence of local progression. The distinction from recurrence or benign lung fibrosis has constantly been difficult. Suspicious images were considered as local relapses without central review. Histological confirmation was not a prerequisite for classifying the lesion as recurrence. Differentiation between benign radiographic changes and local recurrence after SABR remains challenging because many patients developed radiation-induced fibrosis in the treated lung region [18, 19]*.* Therefore, it is likely that some local relapses were actually radiation-induced fibrosis. Regional recurrence was defined as a failure in the hilum, mediastinum or supraclavicular fossae while distant recurrence as a failure in other sites.

### Statistical analysis

Median follow-up was assessed by the reverse Kaplan-Meier method. OS was defined from the start of radiotherapy to death by any cause. DFS was assessed from the start of radiotherapy until death or local, regional or distant relapse. OS and DFS were computed with Kaplan-Meier method. Cumulative incidence function (CIF) of local, regional, distant or any recurrences were computed using competing risk analysis with death as a competing event.

Exploratory subgroup analyses were performed comparatively on OS, DFS and relapses according to 3 factors: treatment period (before 2010 versus after 2010), previous (if any) cancer history (presence versus absence), and tumor stage (T1a versus T1b and T1a versus T2). Cox proportional hazards regression models were used to assess the association between OS and DFS rates and these variables. Fine and Gray’s proportional subdistribution hazards regression models and Cox proportional cause-specific hazards models were used to estimate the subdistribution hazard ratios (sHR) and the cause-specific hazard ratios (csHR) respectively.

The R software version 3.3.2 (The R Foundation for Statistical Computing, Vienna, 2016) was used for statistical analyses with the following packages: *ggplot2*, *survival* and *survminer*.

## Results

### Patient characteristics

One hundred and eighty-nine patients treated with SABR for a single primary lung lesion and also treated with a 12 Gy × 4 fractions scheme were analyzed in this retrospective analysis.

The median age was 72 ± 9,8 (range: 46–89 years), 66% were men, 93% were smokers or ex-smokers. Diagnosis was histologically confirmed in 42% of the patients (*N* = 80), including 21% (*N* = 39) lung adenocarcinoma, 15% (*N* = 29) lung squamous cell carcinoma and 6% (*N* = 12) not otherwise specified NSCLC. The remaining 109 patients were treated without histological confirmation (58%) due to a contraindication for a transthoracic biopsy (e.g. severe COPD status in most of the cases).

The median tumor diameter was 18 mm ± 7,8 (range 6–37 mm). Tumors were located in the right lung in 54% of the patients (*N* = 102), including 32% (*N* = 61) in the upper lobe, 5% (*N* = 9) in the middle lobe and 17% (*N* = 32) in the inferior lobe. Tumors were located in the left lung in the upper and lower lobes in 29% (*N* = 55) and 17% (N = 32) of the patients, respectively.

AJCC 7 TNM stage distribution was; T1a: 59% (*N* = 111), T1b: 30% (*N* = 57), T2: 11% (*N* = 21); all patients were N0 and M0.

There were 411 listed contraindications for 189 patients. The most important contraindications to surgery were pulmonary (*N* = 137 and 72%) and/or cardiac disfunctions (*N* = 80 and 42%). Only 15 patients (8%) refused surgery.

Treatment and patient’s characteristics are shown in Table 1.

**Table 1:** Patient characteristics

Number of patients (N)	189
Male/female	66% (*N* = 124) / 34%(*N* = 65)
Age (median and range)	72 ± 9,8 (46–89 years)
T-stage T1a	59% (*N* = 111)
T1b	30% (*N* = 57)
T2	11% (N = 21)
Tumor size (median and range)	18 mm ± 7,8 (6–37 mm)
Histology Unknown	58% (*N* = 109)
Known ADK	21% (N = 39)
SCC	15% (N = 29)
NOS	6% (N = 12)
Right Lung	54% (N = 102)
Upper lobe	32% (N = 61)
Middle lobe	5% (N = 9)
Inferior lobe	17% (N = 32)
Left lung	46% (*N* = 87)
Upper lobe	29% (N = 55)
Inferior lobe	17% (N = 32)

Abbreviations: *ADK* adenocarcinoma, *SCC* squamous cell carcinoma, *NOS* not otherwise specified

### Overall analysis

The median follow-up of surviving patients is 18 months +/− 21 (interquartile range: 9–33 months).

#### Survival

**Fig. 1:**
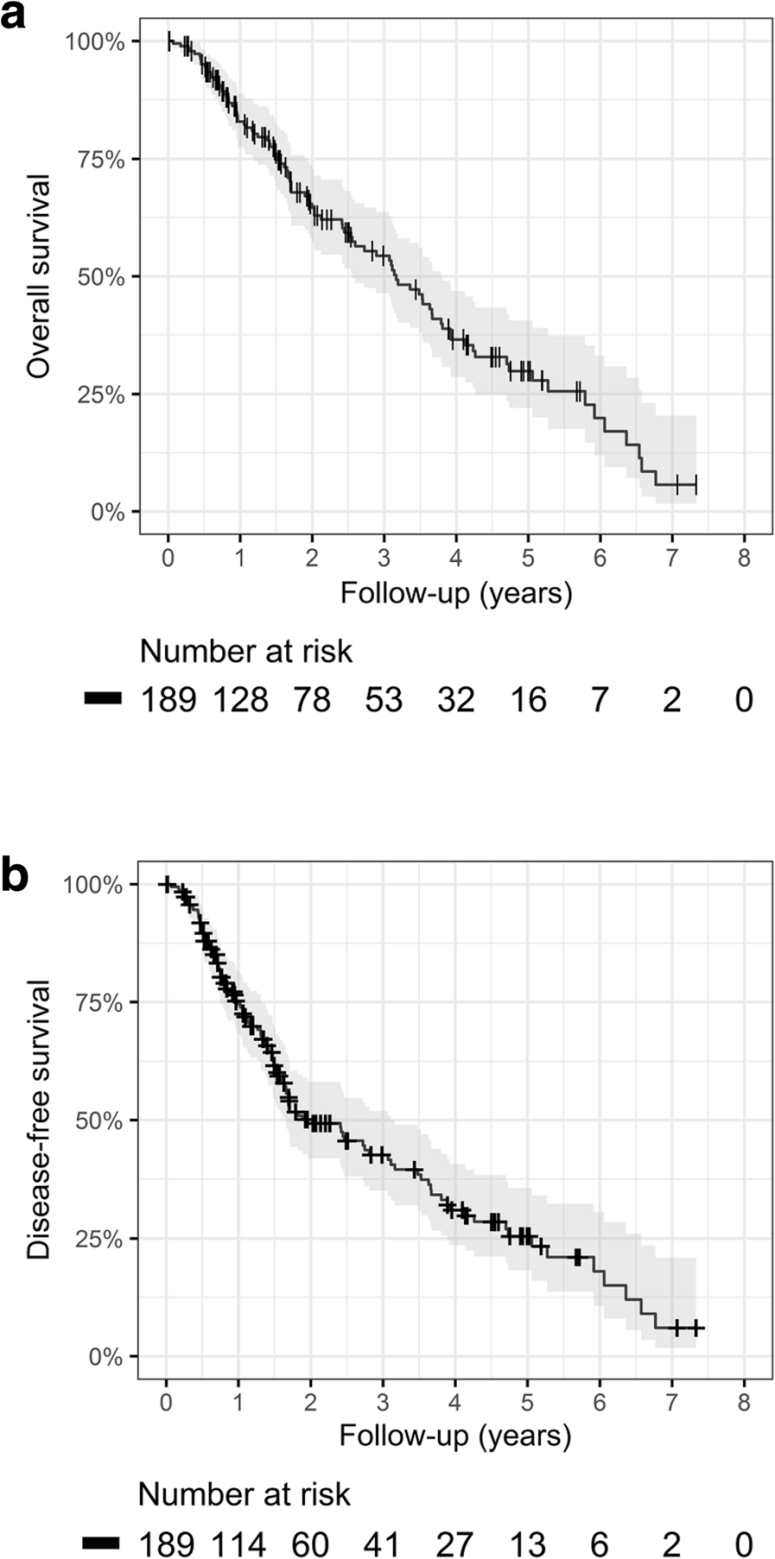
**a** Overall survival (OS) **b** Disease-free survival (DFS)

After 1, 2 and 4 years, the OS rates [95% confidence interval] were 83% [78–89%] (*N* = 128), 65% [57–73%] (*N* = 78) and 37% [29–47%] (*N* = 53), respectively. The median survival time was 37 months. DFS rates were 75% [95% CI: 68–81%] (*N* = 114), 49% [95% CI: 42–58%] (*N* = 60) and 31% [95% CI: 24–41%] (*N* = 41) after 1,2 and 4 years, respectively (Fig. 1a and b).

#### Failure patterns

Overall, we observed 27 patients with local relapses (14%), 18 with regional relapses (10%), 29 with distant relapses (15%) and 51 with at least one relapse (27%).

**Fig. 2:**
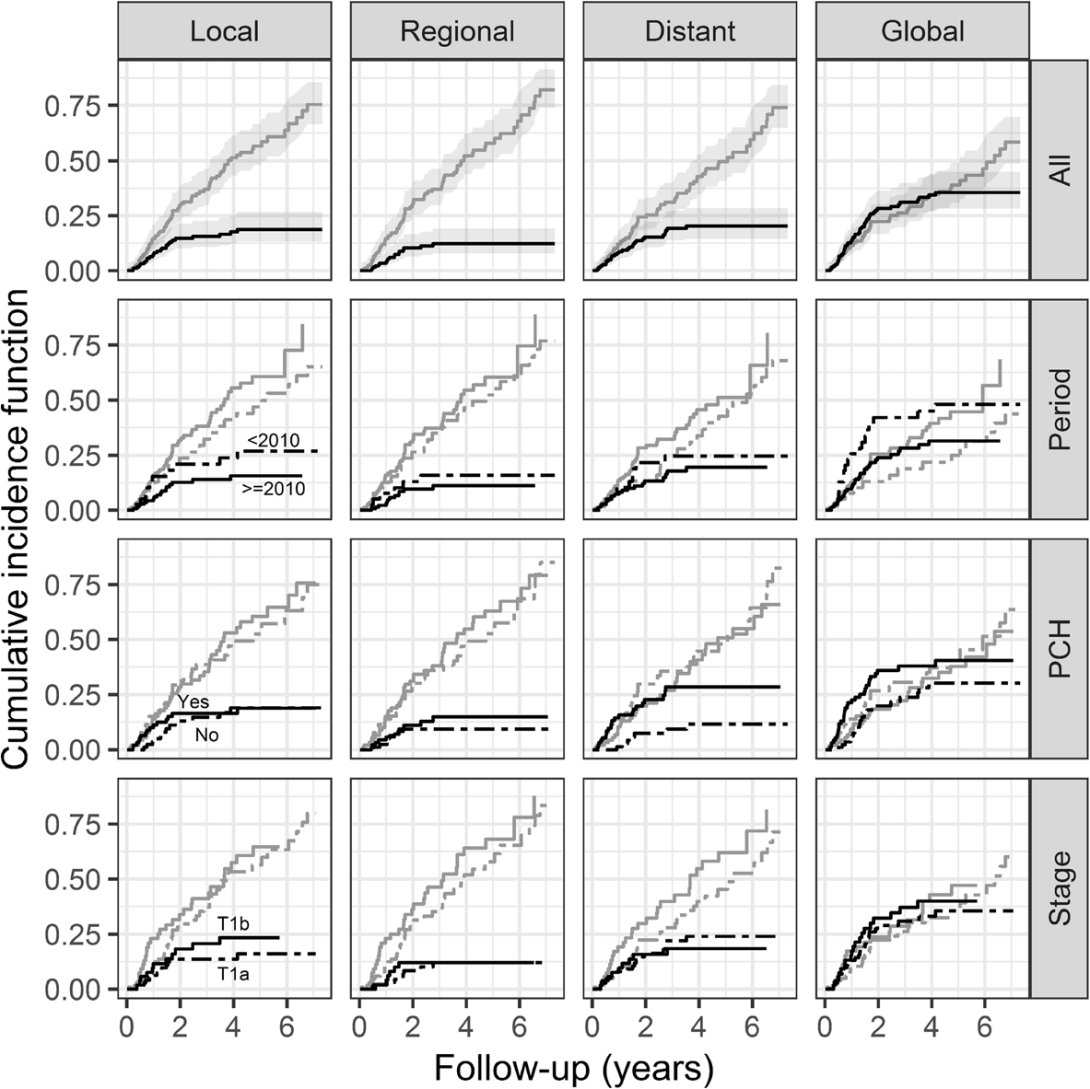
Cumulative incidence function (CIF) of local, regional, distant or any relapses. CIF of relapses and death are represented in black and grey respectively. CIF are presented for the whole group (All), in two groups following the period (before 2010 and after 2010), the existence of a previous cancer history (PCH) and tumor stage (T1a versus T1b, stage T2 is not shown)

At 1, 2 and 4 years, the cumulative incidence function (CIF) of local relapses were 8% [4–13%], 15% [10–21%] and 18% [12–25%], the CIF of regional relapses were 4% [2–8%], 10% [6–16%] and 12% [8–19%], the CIF of distant relapses were 9% [5–14%], 15% [11–22%] and 20% [15–28%] and the CIF of any relapses were 14% [10–20%], 28% [22–36%], 34% [27–43%], respectively (Fig. 2).

One patient was reclassified as locally controlled after a salvage surgery that demonstrated the absence of residual disease.

### Subgroup analysis

Stage T1b (vs T1a) was associated with a worse OS (univariate HR (uHR): 1.49, multivariate HR (mHR): 1.66, 95% confidence interval (CI): [1.06–2.59], *p* = 0.026) and a worse DFS (uHR: 1.43, mHR: 1.57, 95%CI: [1.02–2.41], *p* = 0.038). Treatment period and previous cancer history were not found to be significantly associated with OS nor DFS.

Treatment period was associated with a decrease of incidence of any recurrences (univariate sHR (usHR): 0.42, multivariate sHR (msHR): 0.49 [0.29–0.90], p = 0.02). The association between treatment period and local relapses was slight but inconclusive (usHR: 0.43, msHR: 0.47 [0.21–1.06], *p* = 0.07).

Previous cancer history was associated with an increase of distant relapses (usHR: 3.28, msHR: 3.33 [1.34–8.27], *p* = 0.01). Stage T1b was not associated with incidence of local, regional or distant relapses.

### Toxicity

For all patients, grade 1 to 3 toxicities were: fatigue (41%; *N* = 77), chest wall pain (10%; *N* = 19), dyspnea (7%; *N* = 14), radiation pneumonitis (4%; *N* = 8, including 2% of grade 3), dermatitis (4%; N = 7), cough (3%; *N* = 6), rib fractures (2%; *N* = 3), and esophagitis (1%; N = 1). No grade 4 or 5 toxicity was observed.

## Discussion

This cohort of 189 frail patients covers a decade of early-stage NSCLC patients treated with SABR in a single radiation oncology department with a median follow-up of 18 months. The 2 and 4-year OS and DFS rates reached 65 and 37%, respectively and 49 and 31%, respectively. At 2 and 4 years, the cumulative incidence function (CIF) of local relapses, regional relapses and distant relapses were 15 and 18%, 10 and 12%, 15 and 20%, respectively. The CIF of any relapses at 2 and 4 years were 28 and 34%, respectively. Our results in terms of clinical outcomes are comparatively similar to recently published data. In a recent review of 72 SABR studies, Chi et al. reported a 5-year OS rate of 41.3% after SABR [11].

Regarding toxicities, our data are also coherent with the recent review of Chi et al. with a low incidence of grade 3 radiation pneumonitis and the overall incidence of rib fractures. The incidence of grade 3 radiation pneumonitis was 2% compared to 3.4% in Chi et al. and the incidence of rib fractures for our study against Chi et al. was 2% vs. 3.2%.

The main limitation of our study is its retrospective nature, although all the patients are treated at a single radiation center after referrals from several hospitals.

A second limitation is the absence of a central review of follow-up imaging. However, most follow-up images were reviewed in the local multidisciplinary thoracic oncology board of the patient’s hospital, including a radiologist and/or a nuclear physician. All suspected lesions were counted as local relapses, which can be seen as another limitation with an under-estimated local control. Histological confirmation was not a prerequisite for classifying lesions as recurrence. Differentiation between benign radiographic changes and local recurrence after SABR remains a challenge as many patients developed radiation-induced fibrosis in the treated lung region [18, 19]*.* Therefore, it is likely that some local relapses were actually radiation-induced fibrosis.

Patients treated without histological confirmation had to meet the following criteria: an increase in tumor size over time, a high FDG-PET uptake and morphological abnormalities suggestive of malignancy. When all these criteria are fulfilled and there were no signs of infection, the probability of benign diagnosis is less than 4% [14, 15]. The recent American Society for Radiation Oncology (ASTRO) guideline acknowledges the concept of treating with SABR without histology in this context [20]. A high proportion of patients in our series were treated without histological confirmation (58%). This may indicate a high proportion of frail patients in our population with a contraindication for a transthoracic biopsy.

As compared to the recent international Advisory Committee on Radiation Oncology Practice (ACROP) recommendations, our methods especially since 2010, are in tandem in terms of the radiation preparation, prescription methods, delivery, and technologies [21]. One difference with our group of patients is the recommendation of more frequent use of post-therapeutic biopsies if a relapse is suspected for patients eligible for salvage treatments. This was not the current practice in our patient series, as 58% were already considered ineligible for a transthoracic biopsy during the initial diagnosis. Another major difference with these recommendations is the radiation dose. Our 12 Gy × 4 fractions schema corresponds to a BED with an alpha/beta of 10 in the linear quadratic model (BED 10) of 106 Gy, superior to the favorable 100 Gy threshold. The ACROP consensus proposed to keep the 12 Gy × 4 fractions schema for tumors with a substantial contact with the chest wall, or an adapted 15 Gy × 3 fractions escalated schema (BED 113 Gy), and for the best prognosis patients, a much higher dose of 18 Gy × 3 fractions. Considering our slightly lower than expected local control (74% for the whole patient cohort at the median follow-up of 4 years and 1 year), our prescriptions nowadays are sometimes adapted in accordance to ACROP recommendation (18 Gy × 3 fractions) depending on the clinical situation and when a higher dose can safely be delivered.

The substantial technological advancements during the recruitment of patients can be viewed as another methodological weakness, although they represent real-life practice. The patients were treated with a nominally constant dose prescription. However, considering the fact that the dose was first prescribed at the isocenter using a pencil beam algorithm, and later at the isodose encompassing the PTV using an AAA algorithm, proved a significant dose escalation by at least 25%. The studied outcome parameters were not clearly improved by the new technologies or with the derived prescription method, but we remark a slight association between the treatment period and the incidence of any recurrences. However, the decrease in relapses is concomitant with an increase of deaths. The positive impact of the treatment period must therefore be further demonstrated. The limitations of this non-randomized comparison highlights the difficulties in radiation oncology to assess the potential clinical improvement of new technologies, as illustrated by Chi et al. when trying to compare SABR with hypo-fractionated particle beam therapy [11]. Typically, the financial investment for the new radiotherapy machine and methods (dose computation, arc delivery, volumetric image guidance methods, full breathing cycle gating) were performed in 2010, while the improved clinical outcome (improved local control) was only hypothesized in our 2017 review and is not even confirmed.

## Conclusion

We observed a long-term local control and survival after SABR for peripheral stage I NSCLC in this large series of patients with the expected low toxicity.

## Data Availability

The datasets used and analyzed during the current study are available from the corresponding author on reasonable request.

## Abbreviations

4D-CT: a four-dimensional computed tomography
AAA: analytic anisotropic algorithm
ACROP: Advisory Committee on Radiation Oncology Practice
ADK: Adenocarcinoma
ASTRO: American Society for Radiation Oncology
BED: Biologically effective dose
CBCT: Cone-Beam Computed Tomography
CTCAE: Common Terminology Criteria for Adverse Events
CTV: Clinical target volume
FDG-PET-CT: fluoro-deoxy-glucose positron emission computed tomography
GTV: Gross tumor volume
HU: Hounsfield Unit
ITV: Internal target volume
MLC: Multi-leaf collimator
MV: Mega Voltage
NOS: Not otherwise specified
NSCLC: Non-small cell lung cancer
OAR: Organs at risk
OS: Overall survival
PCH: Previous cancer history
PRV: Planning at risk volume
PTV: Planning target volume
RFS: Relapse-free survival
RPM: Real-time Position Management
SABR: Stereotactic ablative body radiotherapy
SCC: Squamous cell carcinoma
TVs: Target volumes
VMAT: Volumetric modulated arc therapy
VMS: Varian Medical Systems
